# Adenoviral-Mediated Endothelial Precursor Cell Delivery of Soluble CD115 Suppresses Human Prostate Cancer Xenograft Growth in Mice

**DOI:** 10.1002/stem.145

**Published:** 2009-09

**Authors:** Trevor Lucas, Dietmar Abraham, Gerold Untergasser, Karin Zins, Erhard Hofer, Eberhard Gunsilius, Seyedhossein Aharinejad

**Affiliations:** a Laboratory for Cardiovascular Research, Department of Anatomy and Cell Biology, Vienna Medical UniversityVienna, Austria; b Tumor Biology and Angiogenesis Laboratory, Division of Hematology and Oncology, Innsbruck Medical UniversityInnsbruck, Austria; c Molecular Vascular Biology, Department of Vascular Biology and Thrombosis Research, Vienna Competence Center, Vienna Medical UniversityVienna, Austria

**Keywords:** Adenovirus, Cellular therapy, Endothelial cell, Gene delivery systems in vivo or in vitro, Immunodeficient mouse, Peripheral blood stem cells, Progenitor cells, Xenogeneic stem cell transplantation

## Abstract

Prostate cancer tumor growth and neovascularization is promoted by an interplay between migratory tumor stromal cells such as specialized tumor-associated macrophages (TAMs) and circulating endothelial precursor cells (CEPs). As vehicles for tumor therapy, human CEPs are relatively easy to isolate from peripheral blood, are able to proliferate long-term in vitro, are amenable to viral manipulation, and preferentially home to regions of ischemia found in growing tumors. We show here that human peripheral blood CEPs expanded ex vivo migrate to prostate cancer cells in vitro and efficiently home to human prostate tumor xenografts in vivo. Infection of precursors ex vivo with an adenovirus constructed to secrete a soluble form of the colony-stimulating factor-1 receptor CD115 that inhibits macrophage viability and migration in vitro significantly decreases the number of TAMs in xenografts (*p* < .05), reduces proliferation (*p* < .01) and vascular density (*p* < .03), and suppresses the growth of xenografts (*p* < .03). These data show for the first time that targeting stromal cell processes with cellular therapy has the potential to retard prostate tumor growth.

## INTRODUCTION

Tumor development is characterized by complex interactions between cancer cells, tumor stroma cells, and the extracellular matrix (ECM). Neovascularization is essential for primary solid tumors and secondary metastases to grow beyond 1-2 mm [1]. Microvessels may develop either from existing capillary networks by neoangiogenesis and arteriogenesis, from bone marrow (BM)-derived angioblasts, or circulating endothelial progenitors (CEPs) in a process known as vasculogenesis [2].

Specialized stromal mononuclear phagocytes known as tumor-associated macrophages (TAMs) also play a key role in tumor neovascularization [3]. TAMs are derived from a minor subpopulation of circulating monocytes [4] and are proangiogenic in tumor regions of hypoxia [5], promoting ECM remodeling by producing matrix metalloproteases, secreting proangiogenic growth factors such as vascular endothelial growth factor (VEGF), and stabilizing the tumor vasculature [6]. TAM recruitment to solid tumors is strongly mediated by colony-stimulating factor-1 (CSF-1) acting through the receptor tyrosine kinase CD115 (also known as the CSF-1R product of *c-fms*) expressed by TAMs [7]. In breast [8] and prostate [9] cancer, TAM accumulation is associated with poor prognosis and survival. Inhibition of TAM recruitment and survival by targeting CSF-1 and CD115 in breast cancer models has a significant effect on tumor development [10,11].

The capability to transplant autologous, nontransformed cell populations that migrate to sites of tumorigenic development to deliver targeted, antitumor therapy has been a long-term goal of cellular cancer therapy. By targeting biological processes obligatory to tumorigenicity, cellular therapies have the potential to circumvent the development of treatment resistance associated with the instability of cancer cell genomes. Endothelial precursors (EPs) have been isolated from BM, ESCs, and cord blood [12] but circulate in the peripheral blood and can be isolated according to surface marker expression and expanded in an undifferentiated state to therapeutical levels before undergoing senescence [13]. Although the precise role played by EPs to support the neovasculature is controversial [14], EPs are effective at selectively migrating to solid tumors [15,16]. Almost one in six men will be diagnosed with prostate cancer during their lifetime. Although the second leading cause of death from cancer, prostate cancer is a relatively slow-growing malignancy that may be amenable to the applications of cellular therapy [17]. CEPs are increased in the peripheral blood of prostate cancer patients [18], and TAMs also play a crucial role in the development and metastasis of prostate tumors [9]. The potential for viral transduced BM-derived cells to deliver soluble antiangiogenic receptors to tumors has been shown [19]. We therefore hypothesized that human ex vivo expanded CEPs would migrate to human prostate tumors and may be used to inhibit TAM-mediated tumor development by secretion of the extracellular ligand binding domain of CD115.

## MATERIALS AND METHODS

### Generation of an Adenovirus-Expressing Soluble CD115

A plasmid containing mouse CD115 was a kind gift of Professor Richard Stanley (Albert Einstein College of Medicine, New York). A soluble (s) mouse CD115 molecule was constructed by amplifying the normal CD115 open reading frame start consensus in a forward primer (5′-GGAATTCCACCATGGAGTTGGGGCCTCCT-3′) incorporating an EcoR1 linker and truncating the molecule at nucleotide 1530 (GenBank accession no. NM_007779.1) directly proximal to the membrane spanning domain of the open reading frame with a reverse primer (5′-CGGGATCCCGTTACTCATCGGGGAGCTGCTT-3′) incorporating a BamH1 linker. The 2,277-bp construct was gel purified with Qiaex II (Qiagen, Hilden, Germany, http://www1.qiagen.com) and ligated into the EcoR1 and BamH1 sites of the pDNR-CMV donor vector, in which the MCS and distal chloramphenicol resistance gene are enclosed by loxp sites (Adeno-X expression systems 2; Clontech, Palo Alto, CA, http://www.clontech.com) as p(s)CD115 and the reading frame of isolated plasmids was confirmed by sequencing on a PRISM 3,100 Genetic Analyzer (Applied Biosystems, Foster City, CA, http://www.appliedbiosystems.com). The plasmids pDNR and p(s)CD115 (200 ng) were incubated with cre recombinase (100 ng) and recombined into the Adeno-X backbone according to the manufacturer's protocol. The reaction mix was transformed into Electromax DH10B cells (Invitrogen, Carlsbad, CA, http://www.invitrogen.com) in a Genpulser (Bio-Rad, Hercules, CA, http://www.bio-rad.com), recombinants were selected on LB plates containing 7% (wt/vol) sucrose and 30 μg/ml chloramphenicol (Sigma, St. Louis, http://www.sigma.com), and genomic integration was confirmed by polymerase chain reaction. Genomic clones were amplified, the viral inverted repeats were exposed by PacI digestion, and after purification, the adenoviral genome was serum-free transfected into adherent HEK 293 cells with Lipofectamine (Invitrogen) in the presence of Plus reagent for 4 hours and cultivated further in culture medium. After 7-14 days, cytopathic HEK293 cells were centrifuged and resuspended in phosphate-buffered saline (PBS), and cells were lysed by freeze/thaw cycles in an ethanol-dry ice bath/37°C, cleared by centrifugation, and stored at −70°C or used to propagate HEK293 infection. Adeno-X-DSRed2 adenovirus for expressing red fluorescent protein (AdRFP) was obtained from Clontech and propogated in HEK293 (CRL-1573) culture. Viruses were purified with Adeno-X-maxi kits (Clontech), and the infectious units were determined by hexon antibody staining of HEK293 infections (Adeno-X-rapid titer; Clontech).

### Cell Culture, Transfection, Infection, and Conditioned Medium

HEK293, L-929, CRL-2505 prostate cancer, and SW620 colon carcinoma cells (American Type Culture Collection, Manassas, VA, http://www.atcc.org) were maintained in a tissue culture medium (TCM) of Dulbecco's modified eagle medium (DMEM; PAA Laboratories, Linz, Austria, http://www.paa.at), 50 U/ml penicillin, and 250 μg/ml streptomycin (Gibco; Invitrogen) supplemented with 10% fetal calf serum (culture medium), in a fully humidified air atmosphere containing 5% CO_2_ at 37°C. CSF-1-dependent CRL-2470 mouse macrophages (American Type Culture Collection) were routinely cultured in TCM supplemented with 10% L-929 fibroblast conditioned medium as a source of CSF-1 [20]. Human CEPs were isolated from peripheral blood mononuclear cells from healthy donors on type I collagen (Sigma) and expanded in EGM-2 medium (Lonza, Basel, Switzerland, http://www.lonza.com) as described previously [13] for at least 10 population doublings before commencement of experiments. Human CEPs proliferate in vitro for more than 30 population doublings before entering senescence [13]. Subconfluent CEP cultures were infected with AdCntl, AdRFP, or Ad(s)CD115 at a multiplicity of infection of 100 for 24 hours and subsequently labeled with DilC18 (Molecular Probes; Invitrogen). CEP conditioned medium was prepared by replacing the culture medium with serum- and growth factor-free basal medium and collecting supernatants after 48 hours of culture. Plasmids were serum-free transfected in HEK293 cells at semiconfluence with Lipofectamine in the presence of Plus reagent (Invitrogen) for 4 hours, refed with culture medium, and cultured for 48 hours. Cells layers were washed with PBS and incubated for 24 hours in serum-free TCM before isolation of cellular supernatants. SW620 and CRL-2505 conditioned media were produced by cultivating semiconfluent cell layers in 6-well plates for 24 hours in 2 ml of nonsupplemented TCM. All supernatants were aliquoted, flash frozen, and stored at –80°C.

### Migration and Proliferation Assays

CEPs were labeled with DilC18 and resuspended in serum-free TCM, and 10^5^ cells were applied to the top of a modified Boyden chamber (8-μm pore size, Thincert 12-well format; Greiner Bio-one, Frickenhausen, Germany, http://www.gbo.com/en). Migration was assessed after 8 hours to nonsupplemented TCM, conditioned medium, or confluent CRL-2505 or SW620 tumor cell layers in the bottom of the chamber. Cells surviving on tumor cell layers were photographed up to 3 days later with an inverted fluorescence microscope (Nikon Eclipse; Nikon, Düsseldorf, Germany, http://www.nikonUSA.com). CRL-2470 cells were starved of growth factor for 48 hours in nonsupplemented TCM. Serum was removed for 2 hours, and the medium was replaced with conditioned medium from serum-starved HEK293 cells transfected with pDNR or p(s)CD115 or endothelial progenitor cells (EPCs) infected with AdCntl or Ad(s)CD115. Cells were restimulated with the indicated concentrations of recombinant human (rh)CSF-1 (Chiron, Emeryville, CA, http://www.), and proliferation was assessed with the water soluble tetrazolium (WST) assay after 24 hours. CRL-2470 macrophages were starved of growth factor for 48 hours and labeled with DilC18, and 10^5^ cells were applied to the top of a modified Boyden chamber (Greiner Bio-one). Migration was assessed after 8 hours to a confluent CRL-2505 stroma layer in the bottom of the chamber in EPC conditioned medium in the presence of rhCSF-1 as a chemoattractant.

### Western Blotting

Transfected HEK293 cells were serum starved and supernatants collected after 24 hours. Supernatants were Western blotted (50 μl) onto nitrocellulose membranes (Bio-Rad) and incubated with polyclonal rabbit antibody directed against the extracellular domain of mouse CD115 (US Biological, Swampscott, MA, http://www.usbio.com) and horseradish peroxidase-conjugated donkey secondary antibody (Amersham, Little Chalfont, U.K., http://www.gehealthcare.com). Proteins were detected by chemiluminescence (Supersignal-West- Femto; Pierce, Rockford, IL, http://www.piercenet.com) and quantified by Easy Win 32 software (Herolab, Wiesloch, Germany, http://www.herolab.com).

### Tumor CEP Homing In Vivo

All experiments were approved by the Institutional Animal Care and Use Committee at the Vienna Medical University. Male, pathogen-free 5-week-old *nu/nu* (nude) mice (Harlan-Winkelmann, Borchen, Germany, http://www.harlan.com) were weighed and anesthetized (ketamine hydrochloride/xylazine at 55/7.5 mg/kg, i.p.), and 15 × 10^6^ CRL-2505 cells in 150 μl PBS were injected subcutaneously into the left flank. Tumor growth was monitored daily with calipers, and animals with comparable tumors were coded and divided into experimental groups after 10 days. Tumor volumes were calculated as length × width^2^ × 0.5. Mice were anesthetized, and 10^7^ CEP-DilC18 cells were systemically introduced by injection into the left ventricle. Animals were sacrificed 36 hours or 3 or 13 days later, and the tumors were isolated and weighed. Because of tumor homing failure as a consequence of the injection procedure in 13% of cases, tumors were included into groups where tumor CEP-DilC18 levels exceeded 1.5%.

### Fluorescent Activated Cell Sorter Analysis

Tumors sections were rinsed in PBS, minced, mechanically dissociated after collagenase digestion at 37°C for 30 minutes, and passed through a 100-μm cell strainer. Mouse peripheral blood was collected by tail vein puncture into heparin-coated tubes (Greiner Bio One). Erythrocytes were lysed in 155 mM NH_4_Cl, 10 mM KHCO_3_, and 0.1 mM EDTA, pH 8, for 10 minutes at 4°C. Isolated cells and AdRFP-infected CEPs in vitro were washed in PBS, and 10^4^ viable events were analyzed on a FACscan flow cytometer (BD Biosciences, San Diego, http://www.bdbiosciences.com) with an argon laser tuned to 488 nm. Membrane-compromised cells were excluded with 7AAD (BD Biosciences). CEPs were stained with phycoerythrin- and fluorescein isothiocyanate-conjugated monoclonal antibodies against CD14 (Immunotools, Friesoythe, Germany, http://www.immunotools.com), CD31 (BD Biosciences), CD34 (BD Biosciences), CD45 (BD Biosciences), CD133 (Miltenyi Biotech, Bergisch Gladbach, Germany, http://www.miltenyibiotec.com), CD141 (BD Biosciences), CD146 (Chemicon, Temecula, CA, http://www.chemicon.com), and VEGFR2 (R&D Systems, Minneapolis, http://www.rndsystems.com as described previously [13].

### Histology

Tissue samples were fixed in formalin and paraffin embedded. Sections were rehydrated in graded alcohols, and antigen retrieval was performed in a microwave in 0.1 M sodium citrate (pH 6.5). Sections were blocked in PBS supplemented with 5% horse serum and stained with monoclonal rat anti-mouse F4/80 (Abd Serotec, Kidlington, U.K., http://www.abdserotec.com), rabbit anti-human Ki-67 (Thermo Scientific, Fermont, CA, http://www.thermo), rabbit anti-human and mouse von Willebrand factor (vWF; Abcam, Cambridge, U.K., http://www.abcam.com), or irrelevant isotype matched control antibodies (BD Biosciences). Primary antibodies were detected by sequential incubation with appropriate biotinylated secondary antibodies (Vector Laboratories, Burlingame, CA, http://www.vectorlabs.com) and Alexa Fluor 488-conjugated streptavidin (Molecular Probes; Invitrogen) or horseradish peroxidase-conjugated streptavidin (DAKO, Glostrup, Denmark, http://www.dako.com). Slides were rinsed with PBS and stained with 0.1 μg/ml 4′-6-diamidino-2-phenylindole, mounted in Cityfluor, and analyzed on a fluorescent microscope (Zeiss, Thornwood, NY, http://www.zeiss.com). Ki-67^+^ proliferating cells, rabbit anti-human, and mouse vWF (Abcam)-positive capillaries and F4/80^+^ TAMs were counted in 10 consecutive (magnification, ×20) fields per slide, and results are expressed as cells per section.

### Kupffer Cell Isolation and Phagocytosis Assays

Kupffer cell populations were isolated 3 days after CEP injection essentially as described previously [21]. Briefly, mice were anesthetized, the peritoneal cavity was exposed, a 24-gauge cannula was inserted into the vena porta, and the liver was perfused for 2 minutes with Ca^2+^-free Hank's buffered salt solution (PAA), allowing the blood to drain from an incision in the inferior vena cava. After a further perfusion for 30 seconds with DMEM supplemented with 0.163 U/ml collagenase A (Sigma), the liver was removed, minced, and incubated with agitation at 35°C for 10 minutes in DMEM/collagenase, passed through a 100-μm filter (BD Biosciences), and resuspended in DMEM supplemented with 2 μg/ml DNase. Cells (10^6^/well) were seeded in 6-well plates and cultured for 2 hours at 37°C in culture medium, nonadherent cells were removed, and cultures were refed with 3 ml of culture medium supplemented with 1 μl/ml fluorescent latex beads (Sigma) with an average size of 1 μm. Cells phagocytosing more than 10 beads were scored by fluorescent microscopy after 24 hours. Results are expressed as numbers of phagocytes per well in triplicate.

### Statistical Analysis

The Mann-Whitney U-test for unpaired groups was used to compare data between the groups. All statistical tests were two sided. Statistical tests were performed with the use of SPSS software (version 10.0.7; SPSS, Chicago, IL, http://www.spss.com). *P* < 0.05 indicated statistical significance.

## RESULTS

### Isolation, Expansion, and Characterization of Human CEPs

**Figure 1:**
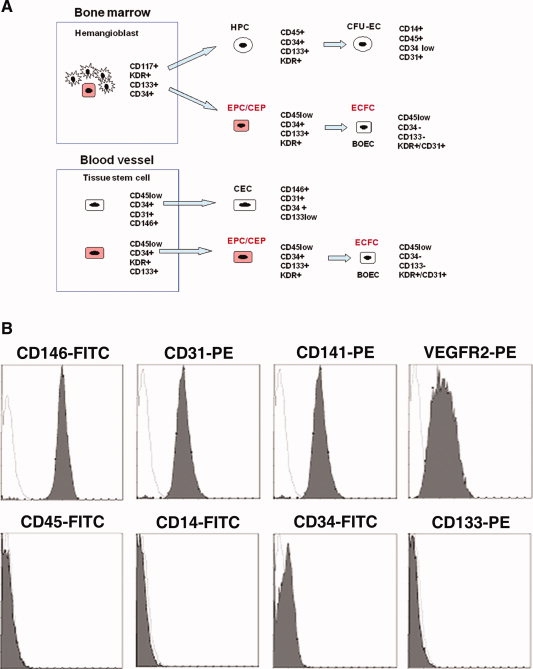
Immunophenotype of human CEPs expanded in vitro. **(A):** Schematic representation of surface antigenic changes in different characterized endothelial cell progenitor populations. Highly proliferative cell lineages are colored red. **(B):** Representative flow cytometric staining of highly proliferative, low and high passage CEPs show stable expression of CD146, CD141, CD31, and VEGFR2, low levels of CD34, and a lack of CD14, CD45, and CD133 antigen expression. Abbreviations: BOEC, blood outgrowth endothelial cell; CEC, circulating endothelial cell; CEP, circulating endothelial precursor cell; CFU-EC, colony forming unit-endothelial cell; ECFC, endothelial colony-forming cell; EPC, endothelial progenitor cell; HPC, hematopoietic progenitor cell.

The human CEPs described here are routinely cultured from peripheral blood mononuclear cells by adherence to type 1 collagen-coated flasks and are characterized by a high proliferative potential and lack of spontaneous differentiation in response to angiogenic growth factors [13]. Distinct from development of circulating endothelial cells (CECs) and the colony-forming unit endothelial cells (CFU-ECs) that also have the potential to adhere in vitro but show limited proliferative capacity, CEPs more closely resemble circulating endothelial progenitors (EPC/CEP), endothelial colony-forming cells (ECFCs), or blood outgrowth endothelial cells (BOECs), shown diagrammatically in Figure 1A; these have been previously described [22,23]. Low- and high-passage CEPs show a stable surface antigenic profile expressing endothelial cell antigens (CD146, CD141, CD31, and VEGFR2) but lacking blood (CD45) or myeloid (CD14) cell markers (Fig. 1B). Cultured CEPs express low levels of CD34 but, in contrast to EPC/CEPs, lack expression of CD133 (Fig. 1B), which is most likely lost during adhesion and propagation.

### Human Ex Vivo Expanded CEPs Migrate to Prostate Cancer Cells In vitro

**Figure 2:**
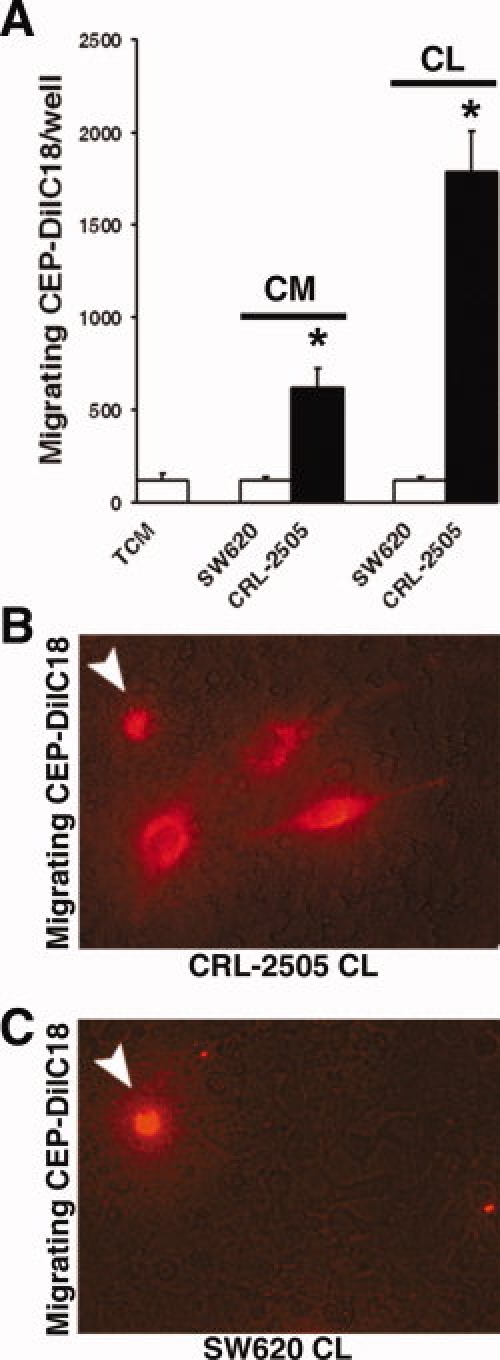
CRL-2505 prostate cancer cells promote CEP migration in vitro. **(A):** CRL-2505 CM and confluent CLs significantly promote the migration of DilC18-labeled CEPs (CEP-DilC18) in vitro compared with SW620 colon cancer cells and serum-free TCM controls (∗, *p* < .001). **(B):** In CL cultures, in addition to CEPs scored in suspension (arrow), a significant number of migrated CEP-DilC18s also extend cytoplasmic processes on the underlying tumor cell layer after 48 hours (magnification, ×20). **(C):** In SW620 CL cultures, only CEP-DilC18s in suspension are observed (magnification, ×20). Abbreviations: CEP, circulating endothelial precursor cell; CL, cell layer; CM, conditioned medium; CL, cell layer; TCM, tissue culture medium.

We initially screened a panel of tumor cell lines and conditioned medium for activities to promote the migration and survival of CEPs in vitro. Summarized in Figure 2A, both CRL-2505 prostate cancer conditioned medium and confluent CRL-2505 cell layers significantly promoted the serum-free migration of DilC18 fluorescently labeled CEPs (CEP-DilC18) in vitro compared with tissue culture medium or a cell line lacking this activity such as SW620 colon cancer cells (*p* < .001). In the serum-free cell layer cultures, 1.14 ± 0.33% (mean of three experiments) of migrating CEPs also extended cytoplasmic processes on the CRL-2505 cell layer (Fig. 2B) not seen in SW620 cells (Fig. 2C). These data show that CRL-2505 prostate cancer cells can promote the migration and serum-free survival of human CEPs in vitro.

### Human Ex Vivo Expanded CEPs Home to Human Xenotransplanted Prostate Tumors In Vivo

**Figure 3:**
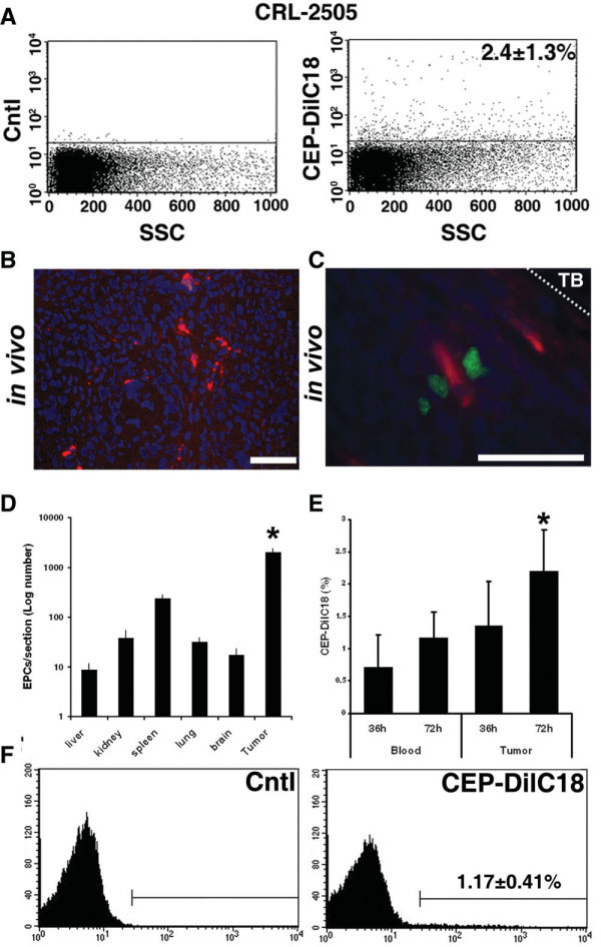
CEPs preferentially home to CRL-2505 prostate cancer xenografts in vivo. CEP-DilC18s were introduced into mice bearing established CRL-2505 xenografts. **(A):** Representative flow cytometric side scatter plot analysis showing that 2.4 ± 1.3% of tumor material is comprised of CEP-DilC18s 3 days after administration compared with control xenografts. **(B):** Representative fluorescence microscopy images of exogenous CEP-DilC18s migrated to xenotransplanted CRL-2505 tumors shows CEP-DilC18s are found throughout the tumor (left panel; magnification, ×40; bar, 20 μm). **(C):** Representative images show that CEP-DilC18s colocalize with tumor cells staining for Ki-67^+^ (Alexa-fluor 488, green; nuclear counterstain, diamidino-2-phenylindole, blue) in the peritumoral space within 20 cell layers of the TB 13 days after injection. **(D):** In major organs, exogenous CEP-DilC18s are primarily found in the tumor (∗, *p* < .001) compared with all other organs after 13 days. **(E):** Detection of CEP-DilC18s in peripheral blood and tumors 36 and 72 hours after injection (∗, *p* = .03). **(F):** A representative fluorescent activated cell sorter histogram showing levels of CEP-DilC18s in peripheral blood after 72 hours compared with control mice. Abbreviations: CEP, circulating endothelial precursor cell; Cntl, control; TB, tumor boundary.

To determine whether human ex vivo expanded CEPs migrate to CRL-2505 in vivo, subcutaneous tumors were implanted in immune compromised athymic *nu/nu* mice for 10 days, and 10^7^ CEP-DilC18 were systemically introduced by intraventricular injection. Shown representatively in Figure 3A, fluorescent activated cell sorter (FACS) analysis of isolated tumor lysates demonstrated that 2.4 ± 1.3% of the tumor was made up of CEP-DilC18s 3 days after injection. The rather large variation in CEP-DilC18 scatter characteristics observed is consistent with the variable morphologies adopted by EPs in the tumor microenvironment [24]. In tissue sections, nucleated CEP-DilC18s were located throughout the growing CRL-2505 tumor at densities corresponding to the FACS analysis data, indicating stability of the DilC18 dye during tissue embedding and fixation (Fig. 3B). Defining the peritumoral space as the first 20 cell layers, CEP-DilC18 were nonuniformly distributed within the tumor. Analysis of serial sections showed that 77.3 ± 7.9% and 74.64 ± 12.3% of tumoral CEP-DilC18s were localized to the actively growing peritumoral region 3 and 13 days after administration, respectively, shown representatively in Figure 3B, in the vicinity of peritumoral Ki-67^+^ proliferating tumor cells (Fig. 3C). We next assessed the tissue distribution of CEP-DilC18s in different organs. Similar to tail vein injection of human EPCs [16], the highest density of introduced CEPs accumulated in tumor tissue (1,991 ± 370 cells/section), significant numbers were found in the spleen (237 ± 44), and only low levels (<37 cells/section) were found in other major organs (liver, kidney, lung, and brain) examined 13 days after injection (Fig. 3D). To study the kinetics of circulation and homing of CEP-DilC18s in the initial stages after injection, we assessed levels in the peripheral blood and tumors 36 and 72 hours after injection by flow cytometry (Fig. 3E, 3F). Data indicate that levels of tumor-associated CEP-DilC18s increase between 36 and 72 hours after injection (*p* = .03). Although differences in CEP levels between time points in peripheral blood did not reach statistical significance, a clear trend toward increased circulating levels could indicate the presence of extravasation processes of the administered CEP-DilC18s.

Together these data show that expanded CEPs preferentially migrate to the actively growing peritumoral region of prostate cancer tumors in vivo and are cleared from the circulation primarily in the spleen.

### Construction and Infection of CEPs with Adenoviruses

**Figure 4:**
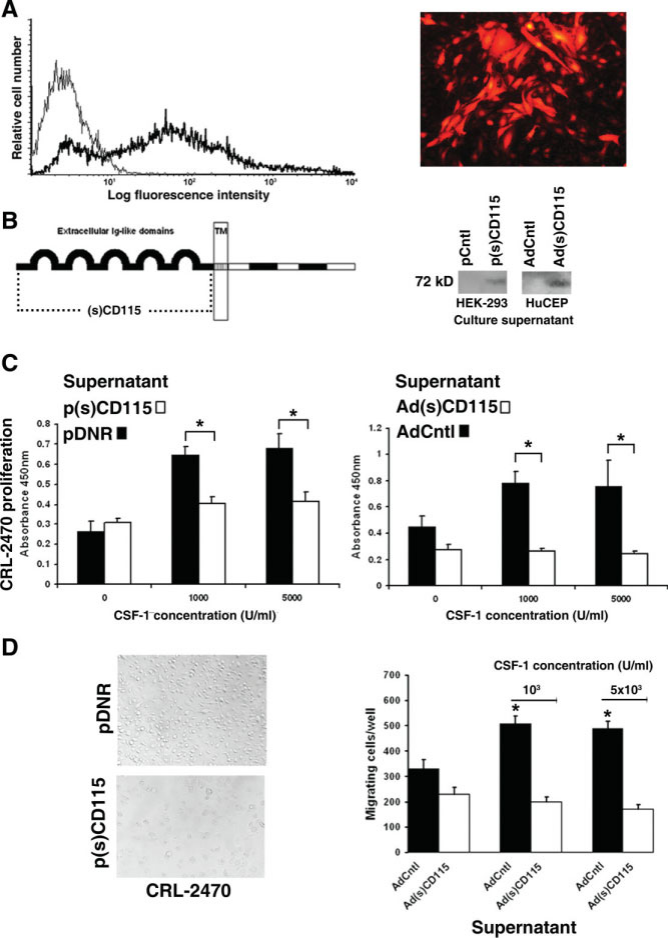
Adenovirus construction and circulating endothelial precursor cell (CEP) infection. **(A):** Marker adenovirus infection of endothelial progenitor cells at a multiplicity of infection of 100 results in expression of red fluorescent protein in more than 74 ± 13% of cells assessed by flow cytometry and fluorescence microscopy. **(B):** An (s)CD115 receptor was constructed by cloning the ligand binding extracellular domains of CD115 proximal to the transmembrane domain (dotted line) into the pDNR donor vector as p(s)CD115 and inserted into an adeno-X adenoviral backbone as Ad(s)CD115. Both p(s)CD115 transiently transfected HEK 293 cells and Ad(s)CD115-infected CEPs secrete the 72-kDa (s)CD115 protein in vitro shown in representative Western blots. **(C):** Serum-free supernatants from p(s)CD115-transfected and Ad(s)CD115-infected cultures significantly inhibit CSF-1-driven proliferation of CRL-2470 mouse macrophages in vitro (∗, *p* < .001). **(D):** Representative images of the effect of p(s)CD115 supernatants on CRL-2470 are shown (magnification, ×20). Serum-free supernatants from Ad(s)CD115-infected CEPs significantly inhibit the migration of CRL-2470 in vitro (∗, *p* < .001). Abbreviations: CSF-1, colony-stimulating factor-1; (s), soluble.

To evaluate the potential of CEP as transient carriers of a potential therapeutic protein in a nonintegrative virus, we assessed the transfection efficiency of CEP with a nonintegrative ΔE1/ΔE3 red fluorescent protein expressing adenovirus (AdRFP) in vitro. As shown in Figure 4A, 74 ± 13% of CEPs are infected by adenoviruses in vitro as assessed by flow cytometric analysis and fluorescence microscopy. Expression of RFP was maintained for up to 13 days in vitro (data not shown). As shown in Figure 4B, CD115 is an integral transmembrane receptor with five immunoglobulin-like extracellular domains dependent for binding of CSF-1 and receptor dimerization and a juxtamembrane domain proximal to transmembrane insertion [25]. A construct containing the leader peptide and extracellular domains of mouse CD115 deleting the transmembrane component and intracellular signaling domains is efficiently secreted by insect cells and effectively binds CSF-1 [26].

To produce a cell therapy CEP-based inhibitor of CSF-1 signaling to specifically target the host stromal compartment, the leader peptide and extracellular domains of mouse CD115 were inserted into the pDNR-CMV donor expression plasmid as p(s)CD115. Transient transfection of HEK293 cells with p(s)CD115 resulted in secretion of the 72-kDa soluble receptor as shown by Western blotting of serum-free culture supernatants (Fig. 4B). After recombination into the adenoviral backbone and viral production in *trans*-complementing HEK293 cells, purified Ad(s)CD115 was used to infect CEPs in vitro. As shown in Figure 4B, CEPs secrete the 72-kDa (s)CD115 receptor after infection with the soluble receptor virus. Secreted protein concentrations were 1.7-fold higher in Ad(s)CD115-infected CEPs than p(s)CD115-transfected HEK293 cells, reflecting increased expression from the adenovirus. Serum-free supernatants from HEK293 cells transiently transfected with p(s)CD115 and CEP infected with Ad(s)CD115 (Fig. 4C) inhibited CSF-1-driven proliferation of mouse CRL-2470 macrophages in vitro at pretitrated, saturating (1,000 IU/ml), and supersaturating (5,000 IU/ml) concentrations of rhCSF-1 (*p* < .001). Representative images of the effect of p(s)CD115 supernatants on CRL-2470 are shown in Figure 4D. Serum-free supernatants from Ad(s)CD115-infecteds CEP also significantly inhibit the CSF-1-dependent migration of CRL-2470 macrophages in vitro (Fig. 4D). These data show that CEPs can be infected to express a soluble CD115 receptor construct from an adenovirus that can suppress the proliferation and migration of macrophages in vitro.

### Cellular Tumor Targeting of TAMs

We next studied whether exogenous CEPs could potentially inhibit TAM recruitment to tumors by infection with Ad(s)CD115. All experiments in vivo were limited to the time span of protein expression from the episomal adenovirus determined in vitro. In preliminary experiments, we showed that native CEPs do not express CD115 in FACS analysis and that infection with Ad(s)CD115 did not influence EPC migration to CRL-2505 cell layers in vitro (data not shown).

**Figure 5:**
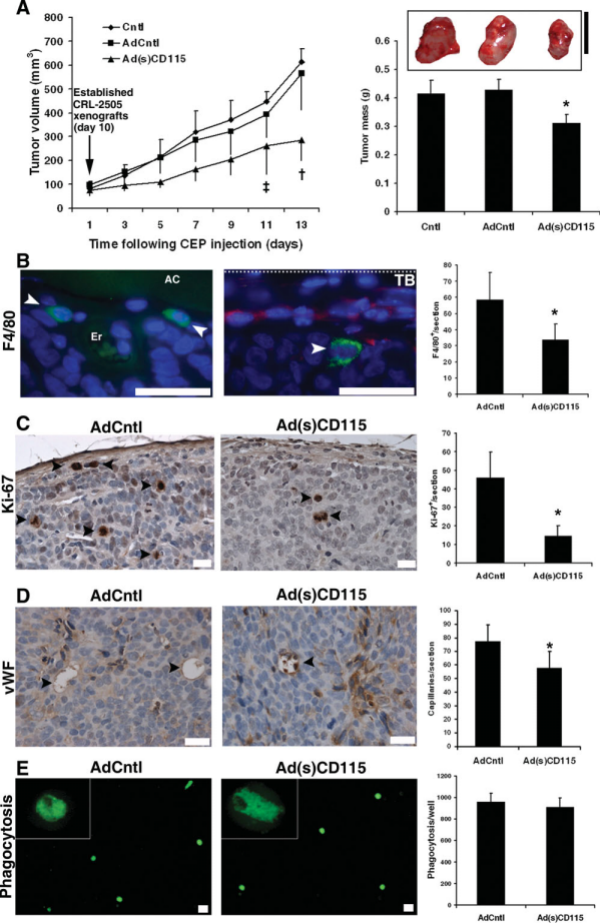
Cellular therapy targeting tumor-associated macrophages. **(A):** Intracardial injection of ringer solution (Cntl) and exogenous CEP-DilC18s infected with AdCntl or Ad(s)CD115 virus significantly influenced the growth of established CRL-2505 xenografts at the indicated time points (^‡^, *p* < .05, ^†^, *p* < .02; data ± SEM) after CEP-DilC18 injection. At termination on day 13 after adenovirus-infected CEP-DilC18 injection, a significant reduction in the tumor masses of CRL-2505 was observed in the Ad(s)CD115 group (∗, *p* < .03 compared with Cntl and AdCntl groups, data ± SEM; bar, 10 mm). **(B):** In CRL-2505 prostate tumors, representative images show TAMs staining positive for F4/80 antigen (Alexa-488, arrowheads) primarily localized in perivascular regions within the tumor in the vicinity of AC areas of extracellular matrix remodeling (left panel; magnification, ×100; bar, 20 μm) or in the peritumoral compartment (right panel; magnification, ×100; bar, 20 μm) in the vicinity of the TB colocalizing with CEP-DilC18. The autofluorescence of erythrocytes denotes the location of a tumoral capillary (nuclear counterstain diamidino-2-phenylindole). Quantification of F4/80^+^ TAM levels shows significant (*p* < .05) decreases in the Ad(s)CD115 group compared with the AdCntl group. **(C):** Representative images (magnification, ×40; bar, 20 μm) and quantification show an associated significant decrease (*p* < .01) in the numbers of proliferating Ki-67-positive cells (arrowheads) in the Ad(s)CD115 group compared with the AdCntl group. **(D):** Representative images (magnification, ×60; bar, 20 μm) of von Willebrand factor staining and quantification show a significant (*p* < .03) reduction in capillary densities in the Ad(s)CD115 group compared with the AdCntl group. **(E):** Kupffer cells isolated from AdCntl and Ad(s)CD115 groups show a similar degree of fluorescent latex bead phagocytosis (magnification, ×4; bar, 10 μm; in vitro) 3 days after CEP administration. Abbreviations: AC, acellular; CEP, endothelial precursor cell; Cntl, control; TB, tumor boundary.

When CEPs were infected with AdCntl or Ad(s)CD115 virus, subsequently labeled with DilC18 and injected into CRL-2505 bearing *nu/nu* mice, CEP migrated to the tumor and organs with similar kinetics to noninfected CEP-DilC18 (data not shown). In the Ad(s)CD115 group, significant reductions in the growth of xenografts were seen from 11 days after CEP injection (*p* < .05 and *p* < .02 between Ad(s)CD115 and both Cntl and AdCntl groups on days 11 and 13, respectively; Fig. 5A, left panel). At termination 13 days after CEP injection, tumor masses (311 ± 97 mg) were significantly (*p* < .03) reduced in the Ad(s)CD115 group compared with the AdCntl (428 ± 85 mg) and Cntl (416 ± 33 mg) groups (Fig. 5A, right panel). No significant differences in the weight of mice between the groups were observed (data not shown).

In CRL-2505 xenotransplants, F4/80^+^ TAMs are detected frequently in perivascular regions neighboring acellular areas of extracellular matrix remodeling (Fig. 5B, left panel) and in the peritumoral region colocalizing with exogenous CEP-DilC18s (Fig. 5B, center panel) of the growing tumor. Assessment of the total number of F4/80^+^ cells (Fig. 5B, right panel) in tumor sections showed a significant reduction (*p* < .05) in TAM densities from 58.6 ± 16.9 to 33.8 ± 9.7 cells/section in the Ad(s)CD115 group compared with the AdCntl group. F4/80^+^ TAM densities were decreased 2.62- and 1.42-fold in the peritumoral and central regions of the tumor, respectively. Accompanying the reduction in tumor growth, Ki-67^+^ proliferating cells were significantly (*p* < .01) reduced in the Ad(s)CD115 group (14.8 ± 5.3 cells/section) compared with the AdCntl group (46 ± 13.8; Fig. 5C). Reductions in Ki-67^+^ cells were observed homogenously throughout the tumor. Assessment of tumor vascularity by counting vWF-positive tumoral vessels (Fig. 5E) distinct from tumoral vWF-expressing CEPs, also detected by the antibody used, additionally showed that TAM reduction was associated with significantly reduced numbers of capillaries throughout the tumor (*p* < .03). These data suggest that, although reduced throughout the tumor, localized effects on TAM densities are increased in the peritumoral compartment where Ad(s)CD115-infected CEP-DilC18s and TAM colocalize, leading to reductions in cancer cell proliferation and vascular density throughout the tumor.

To assess whether Ad(s)CD115-infected CEPs may influence the function of other phagocytes, we isolated Kupffer cells from liver homogenates 3 days after CEP administration. No statistical differences were observed in the ability to phagocytose fluorescent latex beads between the AdCntl- and Ad(s)CD115-infected CEP groups (Fig. 5E). Taken together, these data suggest that (s)CD115-expressing CEPs migrate to prostate cancer tumors in vivo to inhibit TAM levels, reduce tumor cell proliferation, and suppress tumor growth.

## DISCUSSION

In this study, we showed that exogenous ex vivo expanded human CEPs infected with a therapeutic adenovirus specifically migrate to human prostate xenografts in nude mice to suppress tumor growth. The capacity for different EP populations to integrate into the neovasculature in regions of ischemia of solid tumors has varied between different studies [27]. Syngenic BM-derived EPs incorporate into the vasculature of Lewis lung carcinoma [28] and are responsible for the angiogenic switch in lung metastasis [29]. In a variety of human cancers, BM-derived ECs have been found to contribute up to 5% of the tumor endothelium [30]. More recently, a more perivascular role for CEPs has been shown in a variety of mouse models [14]. Differences in results between studies may in part be attributed to the degree of fractionation and culture of studied EP populations from embryonic, BM, cord, and peripheral blood origins, combined with myeloablation protocols to suppress endogenous BM-derived EP homing. However, in almost all models, EPs have been shown to selectively migrate to tumors.

In studies using exogenous in vitro expanded human EPCs in mouse models, tail vein-injected EPCs form vascular structures in autologous tumors [15] and orthotopic human glioblastoma in SCID mice [16]. Endothelial colony-forming cells isolated by adherence to type I collagen also form functional, chimeric microvessels in fibronectin/collagen implants in NOD/SCID mice [22]. In this study, no evidence was found of exogenous human EPC incorporation into the host *nu/nu* mouse vWF^+^ tumor neovasculature (data not shown), but, similar to human glioblastoma [16], CEPs homed primarily to the spleen and the growing tumor, preferentially localizing to the peritumoral region. Although discrepancies between studies may be caused by different mouse models or of i.v. application routes, these results have relevance for any potential therapeutical utilization of CEP homing to tumors because administered CEPs could potentially contribute to the neovasculature and promote tumor growth.

CEP transplantation can support therapeutical postischemic neovascularization in a variety of tissues [31]. Coinjection of EPC with cancer cells also increases tumor growth in mice [32] and is associated with stabilization of the neovasculature that can be reversed by prior EPC irradiation [33]. No reports have shown to date that systemically administered EPCs influence tumor development, and migrated CEPs in this study did not promote the growth of the established prostate tumors compared with untreated controls. Homing of administered CEPs would be expected to compete with endogenous BM-derived CEPs in the later stages of tumor development [28], which may not be the case in coinjection experiments.

The ease of isolation, self-renewal in vitro, amenability to infection with viruses, and tumor homing capacity of EPCs have been proposed as vehicles for targeted tumor therapy [34]. Embryonic EPs have been used as cellular therapy carriers to locally deliver a 5-fluorouracil prodrug to lung metastasis [35]. Homing of unfractionated BM-expressing soluble VEGFR2 as an angiogenesis inhibitor suppresses tumor growth [19], although expression of such a soluble receptor could theoretically affect the VEGF-dependent tumoral migration of EPCs [15].

An additional process pivotal to the growth of tumors such as prostate cancer is the migration and infiltration of proangiogenic TAMs [9]. TAMs are a distinct subpopulation of mononuclear phagocytes derived from circulating Tie-2-expressing monocytes that express CD115 [4]. Strategies to target TAMs in prospective cancer therapy [36] have included directly targeting TAM legumain expression with a DNA vaccine [37], the use of chemokine receptor antagonists [38], and TAM re-education from M2 type macrophages to a more proinflammatory, tumoricidal phenotype [39]. Monocyte and macrophage chemotaxis and survival is strongly regulated by the growth factor CSF-1 produced by a variety of cells including fibroblasts, mononuclear phagocytes, and prostate cancer cells [40,41], and reducing intratumoral and systemic CSF-1/CD115 has been shown to suppress breast cancer growth [10,11]. In this study, we showed the efficacy of adapting this strategy for the potential treatment of prostate tumors with cellular therapy delivering a soluble CSF-1 receptor antagonist with a nonintegrative viral strategy to locally reduce TAM levels.

A number of clinical trials have been conducted with therapeutical EPs and different fractions of mononuclear cells. To date, trials have primarily focused on the direct administration of EPs to myocardial infarcts by intracoronary injection or i.m. application for the treatment of critical limb ischemia [42]. More recently, i.v. infusion of autologous EPs has also been shown to be a feasible and safe methodology in the treatment of patients with idiopathic pulmonary arterial hypertension [43].

## CONCLUSION

In this study, we showed that, by directly influencing the localized accumulation of TAM with a soluble CSF-1 decoy receptor expressed by transfusion of exogenous CEPs, a reduction in the proliferative index and vascularity of tumors is accompanied by direct suppression of prostate cancer growth. These data may therefore lead to the use of CEPs in the development of tailored, nontoxic, cellular therapy strategies to combat the growth and development of prostate cancer by inhibiting the localized accumulation of TAM.

## DISCLOSURE OF POTENTIAL CONFLICTS OF INTEREST

The authors indicate no potential conflicts of interest.
